# Imaging findings of ossifying fibromyxoid tumor with histopathological correlation: A case report

**DOI:** 10.3892/ol.2013.1170

**Published:** 2013-01-31

**Authors:** SOSHI IDETA, JUN NISHIO, MIKIKO AOKI, TETSURO ISHIMATSU, KAZUKI NABESHIMA, HIROSHI IWASAKI, MASATOSHI NAITO

**Affiliations:** 1 Departments of Orthopaedic Surgery, Faculty of Medicine, Fukuoka University, Fukuoka 814-0180, Japan; 2 Pathology, Faculty of Medicine, Fukuoka University, Fukuoka 814-0180, Japan

**Keywords:** ossifying fibromyxoid tumor, magnetic resonance imaging, computed tomography, bone scintigraphy

## Abstract

Ossifying fibromyxoid tumor (OFMT) is a soft tissue tumor of uncertain lineage that most often arises in the extremities of adults. Imaging findings of this uncommon tumor are rare. We, herein, present a case of OFMT occurring in the left thigh of a 36-year-old male. Radiological examinations revealed a well-circumscribed subcutaneous mass with an incomplete shell of peripheral ossification, suggesting a benign condition. Following complete excision, the mass was histopathologically diagnosed as an OFMT. The patient demonstrated no evidence of local recurrence within 11 months of follow-up. We describe the clinicopathological and radiological features, and review the relevant literature.

## Introduction

Ossifying fibromyxoid tumor (OFMT) is a rare, recently identified soft tissue tumor of uncertain histogenesis (1). OFMT predominantly arises in the subcutaneous tissue of extremities, and the median age of OFMT patients is ∼50 years (2–4). Clinically, the tumor often presents as a small, slow-growing, painless, well-defined mass that displays a peripheral incomplete ring of ossification on radiography. Histopathologically, OFMT consists of uniform round, ovoid or spindle-shaped cells arranged in nests and cords, and deposited in a variably fibromyxoid stroma. The biological behavior of this tumor varies. Recently, Graham *et al*(4) demonstrated that histopathologically malignant OFMTs exist. OFMT may be mistaken for a number of benign and malignant conditions, including myositis ossificans, ossifying hematoma, tumoral calcinosis, extraskeletal chondroma, low-grade fibromyxoid sarcoma, synovial sarcoma and extraskeletal or parosteal osteosarcoma (5). In this study, we describe the imaging findings of an OFMT occurring in the subcutaneous tissue of the thigh. We also review the cytogenetic and molecular cytogenetic features of OFMT, as well as its clinicopathological characteristics.

## Case report

**Figure 1:**
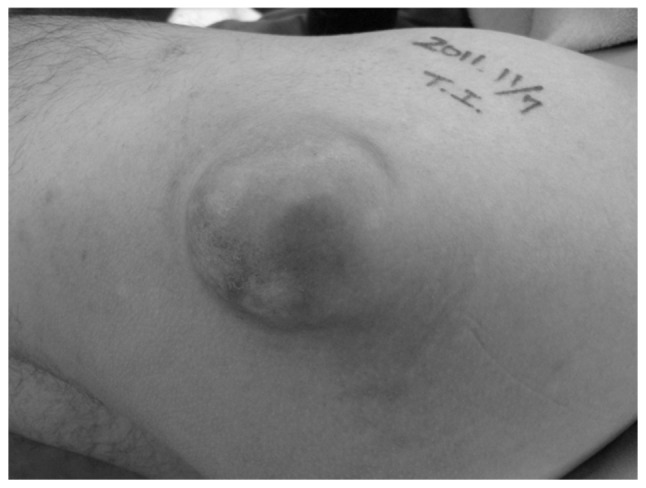
Photograph showing the ∼6×5-cm superficial mass in the posterolateral aspect of the left proximal thigh.

A 36-year-old male presented with a 10-year history of a slow-growing, painless mass in the left proximal thigh. The patient’s medical history was non-contributory. Written informed consent for publication was obtained from the patient. Physical examination revealed a ∼6×5-cm superficial, firm, non-tender mass in the posterolateral aspect of the left proximal thigh (Fig. 1). Neurological and vascular examinations were unremarkable, while the laboratory findings were within normal limits.

**Figure 2:**
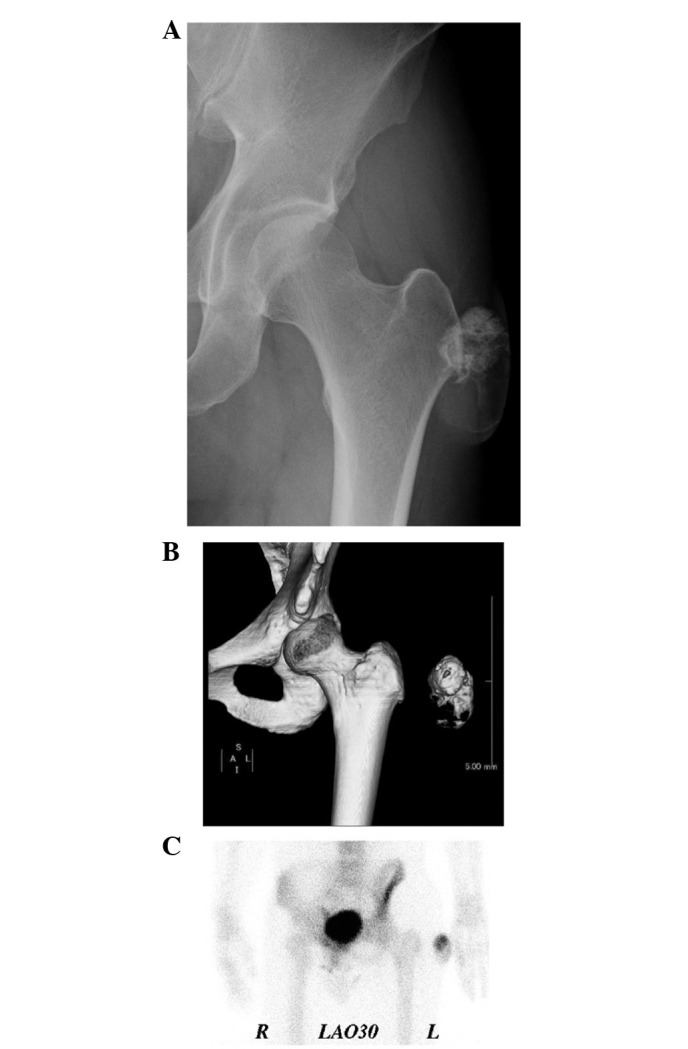
(A) Plain radiograph reveals a soft tissue mass with amorphous calcification and extensive foci of ossification. (B) Three-dimensional computed tomography image shows the presence of the lesion and the normal appearance of the proximal femur. (C) Technetium-99m hydroxymethylenediphosphonate bone scintigraphy reveals heterogenous uptake in the lateral soft tissue of the left proximal thigh.

**Figure 3:**
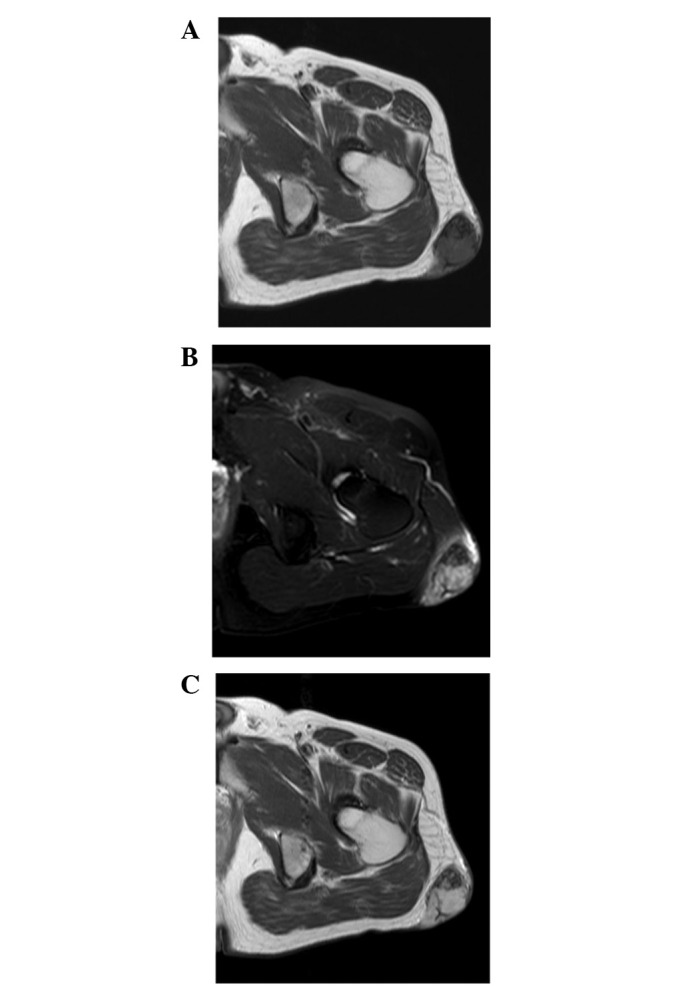
Axial magnetic resonance images of ossifying fibromyxoid tumor in the left proximal thigh. (A) The T1-weighted sequence shows that the mass has low to intermediate signal intensity. (B) The T2-weighted spectral presaturation with inversion recovery sequence shows that the mass has heterogeneous high signal intensity with foci of low signal intensity. (C) The contrast-enhanced T1-weighted sequence shows heterogenous enhancement throughout the mass.

Plain radiographs showed a soft tissue mass with amorphous calcification and extensive foci of ossification (Fig. 2A). Computed tomography (CT) images demonstrated and confirmed an incompletely ossified shell in the lesion (Fig. 2B). Technetium-99m hydroxymethylenediphosphonate bone scintigraphy demonstrated heterogenous uptake in the lateral soft tissue of the left proximal thigh (Fig. 2C). Magnetic resonance imaging (MRI) revealed a well-defined subcutaneous mass. The mass exhibited low to intermediate signal intensity on T1-weighted sequences (Fig. 3A) and heterogeneous high signal intensity, with foci of low signal intensity, on T2-weighted spectral presaturation with inversion recovery sequences (Fig. 3B). A fine linear low signal was observed on both T1- and T2-weighted sequences. Contrast-enhanced T1-weighted sequences demonstrated heterogenous enhancement throughout the mass (Fig. 3C). A benign soft tissue tumor with pronounced ossification was clinically suggested, and the lesion was marginally excised.

**Figure 4:**
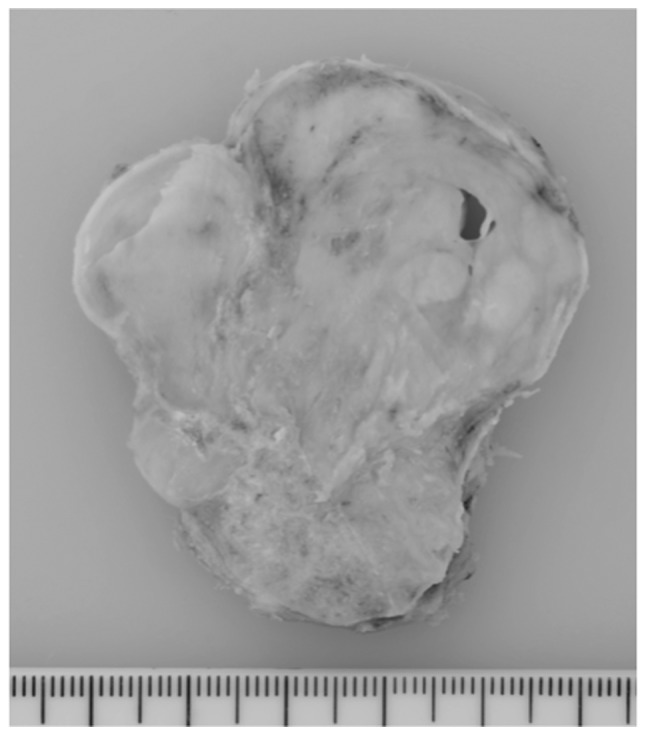
Cut section of the ossifying fibromyxoid tumor displaying a well-circumscribed, gray-white, multinodular appearance.

**Figure 5:**
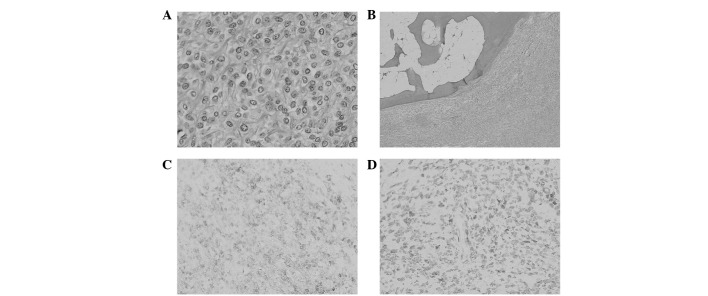
Histopathological and immunohistochemical findings of ossifying fibromyxoid tumor. (A) The tumor is composed of relatively uniform round to oval cells with eosinophilic cytoplasm. (B) A peripheral shell of mature lamellar bone is evident. The tumor cells are immunoreactive for (C) S-100 protein and (D) neuron-specific enolase.

Grossly, the tumor was well circumscribed and covered by a thin fibrous capsule. A cut section revealed that the tumor was gray-white, solid, multinodular and rubbery to firm in consistency (Fig. 4). Histopathologically, the tumor was composed of uniform oval, polygonal or spindle-shaped cells, accompanied by an abundant fibromyxoid stroma (Fig. 5A). Ossification-forming bone shells were found in the periphery of the tumor (Fig. 5B). Necrosis and vascular space invasion were not observed. Immunohistochemically, the tumor cells were positive for vimentin, S-100 protein (Fig. 5C) and neuron-specific enolase (Fig. 5D), and focally for smooth muscle actin and CD56. Immunostaining for epithelial membrane antigen, cytokeratin, desmin, glial fibrillary acidic protein, chromogranin A, synaptophysin and CD57 was negative. The MIB-1 labeling index was <1%. Based on these features, the tumor was diagnosed as an OFMT.

The postoperative course was uneventful, and the patient was doing well with no local recurrence 11 months following surgery.

## Discussion

The majority of OFMTs are clinically and histopathologically benign; however, it has been noted that a subset of OFMTs have atypical histopathological features and exhibit correspondingly more aggressive clinical behavior (2,4,6). In 2003, Folpe and Weiss (2) proposed that OFMT may be classified as typical, atypical or malignant on the basis of its cellularity, nuclear grade and mitotic activity. More recently, Graham *et al*(4) confirmed the existence of malignant OFMT using immunohistochemistry and gene expression profiling. In view of the low to moderate cellularity, low nuclear grade, low mitotic activity and the absence of necrosis or vascular space invasion, our case was regarded as a typical OFMT.

The histogenesis of OFMT remains uncertain. Graham *et al*(4) recently suggested that this tumor exhibits a scrambled phenotype. Typically, as in our case, the cells are positive for vimentin and express S-100 protein. Atypical or malignant areas are observed to express S-100 protein less frequently than the typical areas (2). Other useful markers are CD10 and EAAT4 (3,4).

To date, a limited number of imaging findings of OFMT have been described in detail in the literature (5,7–10). Plain radiographs typically reveal a non-specific soft tissue mass with an incomplete rim of ossification. Erosion or periosteal reaction of the underlying bone is rarely observed. CT scans clearly demonstrate the presence of surrounding or intralesional ossification (7). The MRI appearances of OFMT are variable. The lesion is isointense to muscle on T1-weighted images and shows intermediate-to-high signal intensity on T2-weighted images. There are high signal intensity areas on T1- and T2-weighted images, suggesting hemorrhage and implying a high degree of vascularity (8,9). In addition, areas of ossification demonstrate low signal intensity on T1- and T2-weighted images. As with our case, the ossific element of OFMT has osteoblastic activity that is detected on bone scintigraphy (5,9,11).

Only seven cases of OFMT have been cytogenetically described (2,12–15). Clonal abnormalities of chromosome band 6p21 are prominent. Notably, a balanced or unbalanced t(6;12) (p21;q24) translocation appears to be characteristic for OFMT. A recent fluorescence *in situ* hybridization (FISH) study by Graham *et al*(4) revealed *INI-1* deletion in 71% of cases. Most recently, Gebre-Medhin *et al*(15) demonstrated that *PHF1* (at 6p21) is frequently rearranged in OFMT, including atypical and malignant variants. Moreover, *PHF1* was fused to *EP400* (at 12q24) in one atypical case with the t(6;12) translocation. OFMT is the second neoplasm to be identified, in addition to endometrial stromal tumor, in which *PHF1* is involved in fusions with ectopic sequences. A FISH assay for *PHF1* rearrangements would therefore be useful for the differential diagnosis of OFMT and its histopathological mimics (16).

In summary, we have described the imaging findings of a typical OFMT with clinical and histopathological correlation. Although rare, OFMT ought to be considered in the differential diagnosis of a well-circumscribed, slow-growing, painless, subcutaneous mass with irregular ossifications and/or calcifications.
